# Predictors of Portuguese teachers’ use of Information and Communication Technologies in literacy classes

**DOI:** 10.3389/fpsyg.2022.1006713

**Published:** 2022-11-14

**Authors:** Andreia Nunes, Teresa Limpo, São Luís Castro

**Affiliations:** Center for Psychology at University of Porto, Faculty of Psychology and Education Sciences of the University of Porto, Porto, Portugal

**Keywords:** literacy, education, classroom, teachers, information and communication technology, effective use

## Abstract

In the last years, the teaching and learning of literacy has changed due to the development of Information and Communication Technologies (ICT). The use of ICT in the classroom depends largely on teachers, who are the key players in its integration. However, several factors influence teachers’ decisions to use ICT in their classroom, both internal (e.g., self-efficacy) and external (e.g., school support). Indeed, despite the potential benefits of using ICT, not all teachers use them in their teaching practice. In the present study, we examined which are the main factors influencing teachers’ effective use of ICT in literacy classrooms. A total of 125 teachers lecturing Portuguese Language in grades 5–12 participated in this study (*M* = 50.00 years, SD = 7.88; 89% women). Teachers filled in an online survey, comprising sociodemographic questions (*viz.*, age, gender, education, years of teaching experience, teaching level, school type, and geographical area) and four questionnaires related to ICT and teaching. Results showed that effective use of ICT was predicted by both internal (ICT’ self-efficacy and constructivist conception of teaching) and external (lack of access and support, and gatekeepers) factors. These findings may help in the identification of key targets to facilitate the effective use of ICT in literacy classrooms.

## Introduction

Literacy refers to reading and writing skills and is known to greatly influence the knowledge-building process in the classroom (Iinuma, 2016). However, in the last years, the traditional scope of literacy means – including books, magazines, newspapers, or pen-and-paper writing – was expanded due to the development of different modes of communication provided by Information and Communication Technologies (ICT). The development of ICT led to major changes in the teaching and learning of literacy skills. ICT can be used in the classroom as a supportive tool to deliver traditional instruction (e.g., teachers using PowerPoint presentations) or as a pedagogical means to enable student-centered activities, engaging them in constructive and high-order critical thinking (e.g., students using expert systems in their schoolwork; Jonassen, 1996; Sutherland et al., 2009; Ertmer and Ottenbreit-Leftwich, 2013). As students are becoming more and more comfortable with ICT, its use in the teaching of reading and writing has been capturing researchers’ attention. Despite some advantages of digital methods, such as facilitating reading comprehension (e.g., Mangen et al., 2013) or word memorization (e.g., Mangen et al., 2015), digital methods are recognized to be particularly useful in the teaching of literacy. For example, recent studies found supporting evidence for the use of automated systems in the classroom to enhance reading comprehension (Wijekumar et al., 2017) and writing quality (Nunes et al., 2021).

In line with these benefits of ICT, the European Commission (EC) has recently recognized the importance of ICT in education, by publishing a digital education plan aimed to boost the use of ICT for teaching and learning in schools in the European Union (EU; European Commission, 2018). Still, in the same report, the EC identified several barriers to that end, such as lack of physical conditions (e.g., connection to Internet) in schools in the EU, as well as lack of competences and confidence from teachers to use digital tools to support their teaching. Indeed, introducing ICT into classrooms has been difficult due to several factors, such as government policy failure, inadequate funding, lack of educational vision or the apathy and resistance of teachers (Jewitt, 2006). Teachers are often uncomfortable in using ICT as it implies major changes in their current practice, thereby undermining their confidence to effectively use it in the classroom (Robertson and Dale, 2009; Triggs and Sutherland, 2009).

To know the factors influencing ICT use in education is crucial to successfully integrate ICT into literacy instruction and promote their effective use (Riasati et al., 2012). The effective use of ICT has been studied over the years, resulting in several acceptance models, such as the Technology Acceptance Model (Davis, 1989; Venkatesh and Davis, 2000) and the Unified Theory of Acceptance and Use of Technology (Venkatesh et al., 2012, 2016). According to these models, the benefits of using ICT, ease of use, social influence, and technical support are key determinants that may affect the acceptance and effective use of ICT (Huang and Kao, 2015; Williams et al., 2015). Although these acceptance models provided valuable information on ICT determinants, they are based on general approaches to ICT acceptance and use across educational, organizational, and health settings, that may fail to grasp the specificities of ICT use in the practice. As a consequence, researchers have sought to identify the specific factors that may hinder teachers’ integration of ICT in their classrooms. Ertmer (1999) described two types of factors: internal factors, which are rooted in teachers’ underlying beliefs about teaching and learning (e.g., beliefs and attitudes about the use of ICT), and external factors, which refer to school-related variables, extrinsic to teachers (e.g., the availability of resources).

Internal factors, namely, teachers’ conceptions of teaching and learning as well as their self-efficacy and attitudes toward ICT, are often perceived as the main factors influencing teachers’ ICT integration in their classroom (Drent and Meelissen, 2008; Blackwell et al., 2013). Teachers conceptions about their preferred ways of teaching and learning can be classified in two approaches, based on distinct methods of teaching: teachers with constructivist conceptions tend to use teaching strategies focused on students as active knowledge makers, whereas those with traditionalist conceptions tend to use conventional lecture methods for teaching and perceive students as passive knowledge makers. Previous research has linked these conceptions with ICT use. It seems that teachers with constructivist conceptions tend to be more open to ICT and use it more often in their teaching practice than teachers with traditionalist views (Ertmer, 2005; Palak and Walls, 2009; Ertmer et al., 2012; Teo and Zhou, 2016). Additionally, teachers with a constructivist conception tend to use ICT in their classroom in a more innovative way (Drent and Meelissen, 2008).

Self-efficacy refers to the beliefs people have in their capabilities to complete a task (Bandura, 1997). As stated by Bandura (1995), these beliefs have a strong influence on human behavior, for example, by affecting the effort put on tasks and the level of accomplishment. The power of self-efficacy beliefs affects the use of ICT. Studies showed that teachers with stronger self-perceptions of ability in using a specific teaching tool reported more willingness to use ICT as well as using it more in the classroom (Pajares, 1992; Liaw et al., 2007; Buabeng-Andoh, 2012).

Two major sets of teachers’ attitudes toward ICT involve their perceived value of ICT and their perceived preparedness for using and integrating ICT into the classroom (Blackwell et al., 2013). Negative attitudes toward the value of ICT and disbelief in its pedagogical value has been associated with an ineffective use of ICT in the classroom, even in schools with good resources (Palak and Walls, 2009; Ertmer et al., 2012). Conversely, teachers with positive and strong beliefs in the value of ICT were able to overcome external barriers, such lack of resources, and use these pedagogical tools effectively (Ertmer et al., 2012). Teachers who believed that ICT can benefit children’s learning also reported more use of ICT (Blackwell et al., 2013). Teachers’ perceptions of preparedness for integrating technology into the classroom also influence ICT use (Blackwell et al., 2013). In particular, having enough time to learn how to use ICT and to successfully train it in the classroom showed up as key factors for teachers to integrate ICT into their practice (Ertmer et al., 2012; Johnson et al., 2016).

External factors influencing ICT integration in the classroom are the availability of ICT in schools and the support from key groups of people, such as school administrators and parents (Blackwell et al., 2013). Early studies of ICT integration focused on the availability of ICT tools, such as computers, in schools (Fisher et al., 1996; Norris et al., 2003). This is a basic requirement for the effective use of ICT in education (Johnson et al., 2016), with some studies relating the frequency of computer use with the number of computers available in the classroom (Norris et al., 2003; Inan and Lowther, 2010a). Even though teachers have a central role in using technology for instructional purposes, other school players – known as gatekeepers – were found to influence teachers’ integration of ICT. For example, school support from the administration was associated with innovative uses of ICT by teachers (Drent and Meelissen, 2008). Moreover, when parents perceive ICT to be challenging and beyond their skills, they seem to be less engaged in children’s learning and, consequently, reduce their engagement with ICT to support learning (Osorio-Saez et al., 2021).

Overall, then, several internal and external factors seem to be relevant to successfully integrate ICT into classroom. However, the majority of evidence came from studies focused on university students and L2 teaching, and failed to address specific school subjects, such as L1 (Xu et al., 2019). The use of ICT provides a great support to mother-tongue classes, by helping teachers to center the teaching and learning process on the student rather than on themselves as well as by motiving learners to reading and writing activities (Riasati et al., 2012). For example, writing through ICT is more attractive to students than pen-and-paper writing, as ICT’s writing is more colorful and flashing and can be integrated with sound and image (Matthewman, 2009).

In the present study, our key question was: Which are the main factors influencing teachers’ effective use of ICT in Grades 5–12 literacy classrooms? We focused on Grades 5–12 because we wanted to specifically target teachers responsible for Portuguese Language classes. These only exist from Grade 5 onwards, since before that one teacher is responsible for all the contents (language, maths, social studies, etc.). Also, previous studies reported more use of ICT by teachers lecturing in upper than lower grades, due to the increasing complexity in the curriculum (e.g., Gorder, 2008). To answer our research question, we examined the degree to which internal and external factors predict Portuguese teachers’ effective use of ICT, after controlling for sociodemographic factors, such as age, teaching experience, and gender, which have been related to ICT use (Venkatesh et al., 2003; Magsamen-Conrad et al., 2015; Parameswaran et al., 2015). In comparison with younger, less experienced teachers (the so-called “digital natives”), older teachers, with more teaching experience, are less familiar and thus less willing to use ICT in their classrooms (Prensky, 2001; Foutsitzi and Caridakis, 2019). Female teachers have also been found to have less confidence and experience than males in the use of ICT in teaching (Zhou and Xu, 2007), even though such difference was not observed in another study (Scherer and Siddiq, 2015). After controlling for these sociodemographic factors, we expected that teacher-related internal factors (*viz.*, conceptions of teaching and learning, ICT’ self-efficacy, perceived value of students’ education, teacher-related constraints), and external factors (*viz.*, lack of access and support, and gatekeepers) would influence the use of ICT (Blackwell et al., 2016; Teo and Zhou, 2016).

## Materials and methods

### Participants

A total of 179 teachers lecturing Portuguese Language in grades 5–12 participated in this study. In the Portuguese teaching system, grades 5–6 correspond to elementary grades (10–12 years old), Grades 7–9 to middle grades (12–15 years old) and 10–12 to high-school (15–18 years old). Fifty-four participants were excluded as they did not finish the full online survey. The final sample included 125 Portuguese Language teachers aged between 25 and 65 years (*M* = 50.00 years, SD = 7.88; 88.80% women). As displayed in Table 1, the majority of these teachers lectured in middle-grade years (55.20% in total; 33.60% were exclusively middle-grade teachers), had a Bachelor degree (73.60%), and taught in public schools (92.80%) in the North of Portugal (55.20%). On average, they had a teaching experience of 24.72 years (SD = 9.73), ranging between one and 45 years. The study was approved by the Ethics Committee of the authors’ institution, and informed consent was obtained from all participants.

**Table 1:** Teachers’ characteristics.

Teachers’ characteristics	*N*	%
Gender	125	100
Male	14	11.20
Female	111	88.80
Education		
Bachelor degree	92	73.60
Master degree	28	22.40
Ph.D. degree	5	4.00
Teaching level		
Elementary School (Grades 5–6)	32	25.60
Middle School (Grades 7–9)	42	33.60
High School (Grades 10–12)	24	19.20
Elementary + Middle + High School	2	1.60
Middle + High School	19	15.20
Elementary + Middle School	6	4.80
School type		
Public	116	92.80
Private	7	5.60
Public/private	2	1.60
Geographical area		
North	69	55.20
Center	33	26.40
South	21	16.80
Madeira Isle	2	1.60

### Materials and procedure

The study was conducted online with the Qualtrics software. The link to participate was disseminated through social networks and email, and was available between September 2019 and December 2020. Participants were asked to respond to sociodemographic questions and five questionnaires related to ICT and teaching. Because these questionnaires were originally published in English, they were previously translated to Portuguese by two Portuguese native speakers fluent in English. After discussion, one version of each questionnaire was achieved and presented to 10 Portuguese Language teachers, who provided us the feedback to achieve the final versions (Supplementary Table S1).

#### Conceptions of teaching and learning

We used Teo and Zhou’s (2016) two-dimensional scale of measuring traditionalist (five items; e.g., “Teaching is simply telling, presenting, or explaining the subject matter”; *α* = 0.79), and constructivist conceptions (e.g., “Students should be given many opportunities to express their ideas”; *α* = 0.79). This questionnaire aims to measure the degree to which assume to be the primary source of knowledge and students are the passive receivers (i.e., traditionalist view), and put students into the center of learning seeing them as active knowledge makers (constructivist views). Answers were given in a scale ranging from 1 (*totally disagree*) to 7 (*totally agree*).

#### Self-efficacy toward the use of unfamiliar ICT

We used the computer self-efficacy scale (10 items; e.g., “I could use the new technology if I had never used a product like it before”; *α* = 0.88; Laver et al., 2012; adapted from Compeau and Higgins, 1995). The original computer self-efficacy scale was modified to be able to be used by older people and people with disabilities, and also to cover a broader range of different ICT. Its main goal is to measure people’s confidence about the use of a new and unfamiliar ICT. Teachers were asked to imagine that they received a new ICT they had never used before, intended to facilitate teaching and learning of their subject. Answers were given in a scale ranging from 1 (*not confident at all*) to 5 (*very confident*).

#### Attitudes toward ICT

The attitudes toward ICT measured internal and external factors regarding ICT integration (Blackwell et al., 2013). Internal factors included two dimensions: one about the value of ICT for teachers, specifically the perceived value of ICT for students’ education, where items described how ICT could be useful to children’s cognitive and social development (five items; e.g., “Technology can improve individualized learning”; *α* = 0.78); and other about internal barriers to ICT integration, specifically the perceived teacher-related constraints, where items described teachers’ lack of preparation for integrating ICT into classroom, including lack of training, time to learn or comfort with ICT (five items; e.g., “Technology use is limited by insufficient or lack of training”; *α* = 0.86). External factors included two external barriers to ICT integration: the perceived lack of access and support, where items described how access to ICT and school’ support limit teachers’ use of ICT in the classroom (three items; e.g., “Technology use is limited by insufficient or inadequate software”; *α* = 0.74); and the perceived gatekeepers, which described two major groups of people with power to limit ICT integration into classroom, namely parents and school administration (two items; e.g., “Technology use is limited by my school’s policy” *α* = 0.81). Answers were given in a scale ranging from 1 (*strongly disagree*) to 7 (*strongly agree)*.

#### Effective use of ICT in the classroom

Following the previous work conducted by Blackwell et al. (2014, 2016), teachers were presented with nine different ICT that could be used in the classrooms for instructional purposes (*viz.*, TV/DVD, laptop/desktop computer, digital camera or video recorder, interactive whiteboard, smartphone, e-reader, tablet, and assistive technologies). For each ICT, they were asked to indicate how often they used them for instructional purposes, using a scale ranging from 0 (*never*) to 6 (*daily*). The final score was computed by averaging the responses for the nine ICTs.

### Data analysis strategy

First, we conducted preliminary analyses to examine descriptive statistics and correlations between all variables. Second, we conducted a stepwise multiple linear regression to examine the contribution of internal and external factors on teachers’ effective use of ICT, above and beyond teachers’ sociodemographic characteristics. In Step 1 we entered the main effects of age, gender, and teaching experience. In Step 2, we added the and external predictors to ICT integration.

## Results

### Preliminary analyses: Descriptive statistics and correlations

Table 2 presents means and standard deviations for all predictors and outcome variables, along with the bivariate correlations between them. The correlations can be organized in two main results: (1) Age is only correlated with one factor, specifically with self-efficacy toward the use of unfamiliar ICT (*r* = −0.25); and (2) both internal (i.e., constructivist conception, self-efficacy, teacher-related constraints) and external factors (i.e., lack of access and support) are correlated with teachers’ effective use of ICT in the classroom.

**Table 2:** Means, standard deviations, and correlations for all variables.

Measures	Correlations										
	1	2	3	4	5	6	7	8	9	10	11
1. Age											
2. Gender^a^	−0.01										
3. Teaching experience	0.89^***^	0.09									
4. Traditionalist conception	−0.04	0.07	0.02								
5. Constructivist conception	−0.06	0.07	−0.06	−0.56^***^							
6. Self-efficacy toward the use of unfamiliar ICT	−0.25^**^	0.07	−0.17	−0.13	23^**^						
7. Value for students’ education	0.06	0.05	0.05	−0.27^**^	0.33^***^	0.13					
8. Teacher-related constraints	0.14	−0.05	0.07	0.28^**^	−0.31^**^	−0.50^***^	−0.15				
9. Lack of access and support	−0.04	−0.04	−0.05	0.25^**^	−0.10	−0.20^*^	−0.20^*^	0.38^***^			
10. Gatekeepers	−0.16	−0.05	−0.14	0.23^**^	−0.21^*^	−0.08	−0.14	0.22^*^	0.19^*^		
11. Effective use of ICT	0.002	0.16	0.03	−0.24^**^	0.30^**^	0.33^***^	0.10	−0.28^**^	−0.28^**^	−0.03	
*M*	50.00	0.89	24.72	2.34	6.07	3.84	4.06	2.49	3.60	2.26	1.87
SD	7.88	0.32	9.73	0.82	0.70	0.62	0.06	0.90	0.91	0.71	0.87

a Dummy coded, 0 = male, 1 = female.

^*^p < 0.05; ^**^p < 0.01; ^***^p < 0.001.

### Regression analysis: Prediction of effective use of ICT

Step 1 showed no main effects of teachers’ characteristics in the effective use of ICT, *R*^2^ = 0.03, *F*(3, 121) = 1.10, *p* = 0.35. The inclusion of the factors of ICT integration in Step 2 resulted in a significant increase in the prediction of effective use of ICT, ∆*R*^2^ = 0.23, *F*_change_ (7, 114) = 5.04, *p* < 0.001. Significant predictors were self-efficacy (*β* = 0.25, *p* = 0.01), constructivist conceptions (*β* = 0.21, *p* = 0.05), lack of access and support (*β* = −0.20, *p* = 0.03), and gatekeepers (*β* = 0.18, *p* = 0.05). The final model explained 26% of the variance in the effective use of ICT (*cf.* Table 3 for complete results).

**Table 3:** Final model with all main effects of age, gender, teaching experience and ICT’s and teaching’ related variables on teachers’ effective use of ICT in their classroom.

Predictors	*B*	SE	*β*	*t*
Age	0.01	0.02	0.11	0.58
Gender	0.38	0.23	0.14	1.67
Teaching experience	0.00	0.02	−0.01	−0.03
Traditionalist conception	−0.10	0.11	−0.09	−0.89
Constructivist conception	0.26	0.13	0.21	1.99^*^
Self-efficacy toward the use of unfamiliar ICT	0.35	0.14	0.25	2.61^*^
Value of ICT for students’ education	−0.09	0.13	−0.06	−0.66
Teacher-related constraints	−0.04	0.10	−0.04	−0.37
Lack of access and support	−0.20	0.09	−0.20	−2.26^*^
Gatekeepers	0.22	0.11	0.18	2.03^*^

*R*^2^ = 0.26, *F*(7, 114) = 5.04, *p* < 0.001. ^*^p < 0.05.

## Discussion

Over the years, ICT has been perceived as bringing value to teaching and learning practice (Iinuma, 2016). Consequently, several scholars and organizations have recommended the inclusion of ICT in literacy learning activities (Iinuma, 2016). In the present study, we aimed to determine the role of several internal and external factors influencing teachers’ effective use of ICT in Portuguese Language classes in Grades 5–12. Overall, we found that the effective use of ICT for instructional purposes in Portuguese Language classes were predicted by ICT’ self-efficacy and constructivist conceptions (internal factors), as well as by lack of access and support and gatekeepers (external factors). These findings align with prior research showing that both teacher and school-related factors affect the effective use of ICT in the classroom (Drent and Meelissen, 2008; Inan and Lowther, 2010b; Ertmer et al., 2012). In what follows, we discuss in-depth each of the predictors.

As expected, we found that the endorsement of constructivist conceptions of teaching and learning, but not traditionalist ones, was associated with a greater use of ICT in the classrooms. These findings replicate past research (Palak and Walls, 2009; Teo and Zhou, 2016) and gives strength to the claim that current educational practices need to be changed for ICT to be implemented in a pedagogical way. Foutsitzi and Caridakis (2019) argued that increasing teachers’ familiarity with ICT alone is not enough. Instead, the authors proposed that training teachers on the use of ICT along with pedagogical strategies will allow the teaching and learning process to become a student-centric procedure, in which ICT is used to support students’ exploration and critical thinking. On the same vein, Triggs and Sutherland (2009) argued that ICT needs to go beyond a passive use, in which students only receive information through ICT, to a more learner-centered approach, in which students autonomously manage their engagement with ICT.

In line with our hypotheses, self-efficacy to use technology also appeared as a relevant predictor of effective use of ICT. Conceptually, self-efficacy is based on beliefs rather than on skills, and these beliefs are responsible for the way people deal with adversity and for their confidence in achieving some goal (Bandura, 1997). In our study, teachers had to imagine they were using an ICT they had never used before, with the goal of facilitating the teaching and learning of their subject. Teachers who believed to be more capable of using this unfamiliar ICT reported more use in classroom. This is aligned with prior research that showed that self-efficacy is a major factor in the decision to use ICT as a teaching tool (Pajares, 1992; Liaw et al., 2007; Buabeng-Andoh, 2012). Self-efficacy as a predictor of using ICT in the classroom is not surprising as perceived self-efficacy affects thought processes, the level of motivation and affective states, and therefore affects behavior (Bandura, 1997).

Besides constructivist views and self-efficacy, only external factors were predictors of ICT use. School-related, external factors were found to predict the use of ICT to support the teaching of literacy. Specifically, the more teachers perceived schools as being a barrier to ICT integration (e.g., lack of technical support or equipment), the less often they used ICT. This finding makes sense to the extent that unless schools offer physical conditions to implement technological solutions in the classrooms, teachers will prefer to use more traditional and familiar methods (Grimes and Warschauer, 2008; Ertmer et al., 2012). Additionally, we also found that the perception that teachers had about parents and school’s policy approval of ICT use predicted the use of ICT in the classroom. This finding, which is aligned with past research (Drent and Meelissen, 2008; Inan and Lowther, 2010b), reinforces the need for schools to offer the best possible conditions for teachers to overcome some obstacles to ICT integration. This includes not only to offer adequate equipment, but also to offer technical support when needed, as well as investing in teachers’ professional development, providing them with ICT training (Triggs and Sutherland, 2009; Johnson et al., 2016).

Contrary to our hypotheses, internal attitudes toward ICT did not predict its use. On the one hand, the perceived value of ICT for students’ education was not related to ICT use, which is not aligned with past research (Blackwell et al., 2016). The different findings may be related to methodological aspects (e.g., school levels, type of ICT, research methods) along with cultural factors. In the future, it would be worthwhile to inspect likely variables moderating the link between teachers’ attitudes and ICT use. On the other hand, teacher-related constraints was another variable that did not predict ICT use. Though contrasting with some studies (e.g., Ertmer et al., 2012), this finding is aligned with the results reported by Blackwell et al. (2013), though a preschool context. The authors suggested this lack of predictive value by teachers’ attitudes may be related to a mismatch between their beliefs and the classroom reality. Teachers may use ICT that is available in the classroom, even if they feel restricted in using it due to personal constraints.

Unfortunately, in EU countries including Portugal, ICT is not yet a fully integrated resource in teaching activities (Peralta and Costa, 2007). Studies in the Portuguese context reported that the use of ICT in education is still low and that ICT did not bring a reconfiguration of the educational process, as technology is only being added to traditional teaching methods (Alves and Rodrigues, 2014; Almeida, 2018). This may be troublesome as education do not seem to be fully adjusted to students’ reality. As put by Lacina and Griffith (2012): “For children of the 21st century, technology is like oxygen – a necessary component of their life” (p. 316). The more we adjust teaching to students’ reality, the more likely we are to succeed in improving their educational and professional outcomes.

The findings reported here should be considered in view of at least four limitations. First, because data were obtained at a single time point and because this study is correlational in nature, causality inferences should be avoided. Additional research is needed to replicate reported results using longitudinal designs. Second, there was a larger representation of women than men in our sample. Future studies should aim to collect larger samples and to add more male teachers to the data. Third, given sample size and because some teachers lectured in several teaching levels, an analysis comparing the different teaching levels was not advisable. Future studies should aim for larger samples to provide further conclusions about the use of ICT at different educational stages. Finally, the study was designed before COVID-19 pandemic and, although data collection started before this period, part of the data collection happened during the pandemic. We do not know the extent to which this circumstance, including the massive use of online learning, affected our findings. Future research should explore the impact of this pandemic in teachers’ perceptions and effective use of ICT in the teaching and learning process.

## Conclusion

The use of ICT can be a major help to develop of literacy skills. However, as shown here, there are several teacher and school-related factors influencing its use. To unravel these factors is a first step to foster the use of technology in educational settings. This was the main contribution of the present study, which provided indications on key factors to target in order to improve the use of ICT in Portuguese literacy classrooms. To effectively introduce ICT into the classroom schools should invest in teachers’ professional development and raise their awareness on the potentials of technology to enhance teaching and learning. To that end, it seems important to nurture teacher’s self-efficacy beliefs, promote constructivist views of teaching and learning, and assure that external conditions to support the use of ICT are in place. In sum, the teaching and learning of literacy would much benefit if teachers saw this process as embedded in the digital age.

## Data availability statement

The raw data supporting the conclusions of this article will be made available by the authors, without undue reservation.

## Ethics statement

The studies involving human participants were reviewed and approved by Ethics Committee of the Faculty of Psychology and Education Sciences of University of Porto. The patients/participants provided their written informed consent to participate in this study.

## Author contributions

AN, TL, and SC designed the study. AN implemented the study, analyzed the data, and wrote the first version of the manuscript, under TL and SC supervision. All authors contributed to the article and approved the submitted version.

## Funding

This work was supported by the Portuguese Foundation for Science and Technology (grant UIDB/00050/2020 awarded to the Center for Psychology at University of Porto; Ph.D. studentship SFRH/BD/139195/2018 awarded to AN).

## Conflict of interest

The authors declare that the research was conducted in the absence of any commercial or financial relationships that could be construed as a potential conflict of interest.

## Publisher’s note

All claims expressed in this article are solely those of the authors and do not necessarily represent those of their affiliated organizations, or those of the publisher, the editors and the reviewers. Any product that may be evaluated in this article, or claim that may be made by its manufacturer, is not guaranteed or endorsed by the publisher.
