# Supplementary material for: Predictors of Portuguese teachers’ use of Information and Communication Technologies in literacy classes
Source: Front Psychol. 2022 Nov 14;13:1006713. doi: 10.3389/fpsyg.2022.1006713 (PMC9702811; doi:10.3389/fpsyg.2022.1006713)
Supplement: Supplementary file 1 [file Table_1.docx]

Supplementary Material

Predictors of Portuguese teachers use of Information and Communication Technologies in literacy classes

Andreia Nunes, Teresa Limpo, São Luís Castro^*^

*** Correspondence:** São Luís Castro: slcastro@fpce.up.pt

# Supplementary Table

Table S1: Scales Used, Original English Items and Corresponding Translation to Portuguese.

| Scale |  | Original English Items | Portuguese items |
| --- | --- | --- | --- |
|  |  |  |  |
| Traditionalist Conception  (Teo & Zhou, 2016) |  | During the lesson, it is important to keep the students confined to the textbook and the desks. | Durante as aulas, os alunos devem permanecer sentados nas suas secretárias e utilizar apenas os manuais escolares. |
|  |  | Teaching is simply telling, presenting, or explaining the subject matter. | Ensinar é simplesmente dizer, apresentar ou explicar a matéria. |
|  |  | The traditional/lecture method for teaching is best because it covers more information/knowledge. | Os métodos tradicionais de ensino, de tipo expositivo, são os melhores porque cobrem mais informação/conhecimento. |
|  |  | Teaching is to provide students with accurate and complete knowledge rather than encourage them to discover it. | Ensinar é dar aos alunos conhecimento rigoroso e completo em vez de os encorajar a descobrir por eles próprios. |
|  |  | Good teaching occurs when there is mostly teacher talk in the classroom. | O bom ensino acontece quando é maioritariamente o professor a falar na sala. |
|  |  |  |  |
| Constructivist Conception  (Teo & Zhou, 2016) |  | Every child is unique or special and deserves an education tailored to his or her particular needs. | Cada criança é única e especial e merece uma educação adaptada às suas necessidades particulares. |
|  |  | Good teachers always make their students feel important. | Os bons professores fazem sempre com que os alunos se sintam importantes. |
|  |  | Students should be given many opportunities to express their ideas. | Os alunos deveriam ter muitas oportunidades de expressarem as suas ideias. |
|  |  | Good teachers always encourage students to think for answers themselves. | Os bons professores encorajam sempre os alunos a pensar por si. |
|  |  | Learning means students have ample opportunities to explore, discuss, and express their ideas. | Aprender significa que os alunos têm várias oportunidades de explorar, discutir e expressar as suas ideias |
|  |  |  |  |
| Self-Efficacy Toward the Use of Unfamiliar ICT  (Laver et al., 2012; adapted from Compeau & Higgins, 1995) |  | I could use the new technology… | Indique em que medida se sentiria confiante em utilizar o programa novo. |
|  |  | If there was no one around to tell me what to do as I go | Se não houvesse ninguém à minha volta para me dizer o que fazer. |
|  |  | If I had never used a product like it before. | Se nunca tivesse usado um programa como este antes. |
|  |  | If I had only the product manuals for reference. | Se apenas tivesse o manual de instruções para consultar. |
|  |  | If I had seen someone else using it before trying it myself. | Se, antes de usar o programa sozinho, tivesse visto alguém a utilizá-lo. |
|  |  | If I could call someone for help if I got stuck. | Se pudesse chamar alguém para me ajudar se ficasse bloqueado. |
|  |  | If someone else had helped me get started. | Se alguém me tivesse ajudado desde o início. |
|  |  | If I had a lot of time to complete the job for which the product was provided. | Se tivesse tido tempo para completar a tarefa para qual o programa foi desenvolvido. |
|  |  | If I had just the built-in help facility for assistance. | Se apenas tivesse disponível o menu de ajuda do programa. |
|  |  | If someone showed me how to do it first. | Se alguém me mostrasse primeiro como usar. |
|  |  | If I had used similar products before this one to do the same job. | Se já tivesse usado programas semelhantes para fazer a mesma tarefa. |
|  |  |  |  |
| Value of ICT for Students’ Education  (Blackwell et al., 2013) |  | Technology can improve individualized learning. | A tecnologia pode melhorar a aprendizagem individualizada. |
|  |  | Technology can help to develop children’s critical thinking skills. | A tecnologia pode ajudar a desenvolver o pensamento crítico. |
|  |  | Technology can help to develop children’s higher-order skills. | A tecnologia pode ajudar a desenvolver o raciocínio dos alunos. |
|  |  | Technology can help to develop children’s content knowledge. | A tecnologia pode ajudar a desenvolver o conhecimento dos alunos sobre a matéria. |
|  |  | Technology is useful for social interactions among children. | A tecnologia é útil para as interações sociais entre os alunos. |
|  |  |  |  |
| Teacher-Related Constraints  (Blackwell et al., 2013) |  | Technology use is limited by insufficient or lack of training. | O uso da tecnologia é limitado pela falta de treino ou treino insuficiente. |
|  |  | Technology use is limited by my lack of time to learn technology. | O uso da tecnologia é limitado pela minha falta de tempo para aprender a usar a tecnologia. |
|  |  | Technology use is limited by my lack of time to use technology in my early childhood classroom/program. | O uso da tecnologia é limitado pela minha falta de tempo para usar tecnologia na minha sala de aula. |
|  |  | Technology use is limited by my lack of comfort with technology. | O uso da tecnologia é limitado pelo meu desconforto com a tecnologia. |
|  |  | Technology use is limited because I am unsure of how to make technology relevant to subject areas. | O uso da tecnologia é limitado porque não sei bem como tornar a tecnologia relevante para a minha disciplina. |
| Lack of Access and Support  (Blackwell et al., 2013) |  | Technology use is limited by insufficient or lack of technical support. | O uso da tecnologia é limitado por falta de apoio técnico ou apoio técnico insuficiente. |
|  |  | Technology use is limited by insufficient or inadequate software. | O uso da tecnologia é limitado por software insuficiente ou inadequado (e.g., programas, aplicações, etc.). |
|  |  | Technology use is limited by insufficient or inadequate hardware. | O uso da tecnologia é limitado por equipamento insuficiente ou inadequado (e.g., computador, tablet, wi-fi, etc.). |
|  |  |  |  |
| Gatekeepers  (Blackwell et al., 2013) |  | Technology use is limited by the lack of parent approval of technology in my early childhood classroom/program. | O uso da tecnologia é limitado pela falta de aprovação parental. |
|  |  | Technology use is limited by my school/program’s policy that prohibits technology use. | O uso da tecnologia é limitado pela política da minha escola. |
